# Genomic analysis of human lung fibroblasts exposed to vanadium pentoxide to identify candidate genes for occupational bronchitis

**DOI:** 10.1186/1465-9921-8-34

**Published:** 2007-04-25

**Authors:** Jennifer L Ingram, Aurita Antao-Menezes, Elizabeth A Turpin, Duncan G Wallace, James B Mangum, Linda J Pluta, Russell S Thomas, James C Bonner

**Affiliations:** 1 The Hamner Institutes for Health Sciences, Research Triangle Park, North Carolina 27709, USA

## Abstract

**Background:**

Exposure to vanadium pentoxide (V_2_O_5_) is a cause of occupational bronchitis. We evaluated gene expression profiles in cultured human lung fibroblasts exposed to V_2_O_5 _*in vitro *in order to identify candidate genes that could play a role in inflammation, fibrosis, and repair during the pathogenesis of V_2_O_5_-induced bronchitis.

**Methods:**

Normal human lung fibroblasts were exposed to V_2_O_5 _in a time course experiment. Gene expression was measured at various time points over a 24 hr period using the Affymetrix Human Genome U133A 2.0 Array. Selected genes that were significantly changed in the microarray experiment were validated by RT-PCR.

**Results:**

V_2_O_5 _altered more than 1,400 genes, of which ~300 were induced while >1,100 genes were suppressed. Gene ontology categories (GO) categories unique to induced genes included *inflammatory response *and *immune response*, while GO catogories unique to suppressed genes included *ubiquitin cycle *and *cell cycle*. A dozen genes were validated by RT-PCR, including growth factors (*HBEGF*, *VEGF*, *CTGF*), chemokines (*IL8*, *CXCL9*, *CXCL10*), oxidative stress response genes (*SOD2*, *PIPOX*, *OXR1*), and DNA-binding proteins (*GAS1*, *STAT1*).

**Conclusion:**

Our study identified a variety of genes that could play pivotal roles in inflammation, fibrosis and repair during V_2_O_5_-induced bronchitis. The induction of genes that mediate inflammation and immune responses, as well as suppression of genes involved in growth arrest appear to be important to the lung fibrotic reaction to V_2_O_5_.

## Background

Occupational exposure to vanadium pentoxide (V_2_O_5_) has been associated with an increased incidence of chronic obstructive airway disease and a reduction in lung function [1]. V_2_O_5 _is the most common commercial form of vanadium and is the primary form found in industrial exposure situations [2]. Occupational exposure to V_2_O_5 _occurs during the cleaning of oil-fired boilers and furnaces, during handling of catalysts in chemical plants, and during the refining, processing, and burning of vanadium-rich fossil fuels [3].

We previously reported that V_2_O_5 _causes airway disease in rats that is similar to the pathology of asthma and bronchitis in humans [4]. These pathologic changes include mucous cell hyperplasia, increased airway smooth muscle mass, and peribronchiolar fibrosis. Lung fibroblasts are thought to play a major role in V_2_O_5_-induced airway remodeling *in vivo*, as these cells proliferate around airways following injury and deposit collagen which defines the airway fibrotic lesion [4,5].

Vanadium compounds exert cellular stress via inhibition of protein tyrosine phosphatases (PTPs) in cells [6] and through the generation of reactive oxygen species [7,8]. In particular, vanadium compounds have been shown to stimulate release of H_2_O_2 _in several pulmonary cell types, including alveolar macrophages [9], human lung epithelial cells [10], and human lung fibroblasts [11]. Vanadium-induced oxidative stress has been reported to increase the phosphorylation of MAP kinases through the epidermal growth factor receptor (EGFR) [12] and stimulate activation of multiple transcription factors including p53 [13], AP-1 [14], NF-κB [15] and STAT-1 [8]. These transcription factors play major roles in cell proliferation, apoptosis, differentiation, and the induction of pro-inflammatory mediators. These cellular responses, in turn, determine the overall pathologic outcomes (e.g., inflammation, fibrosis) that lead to the development of V_2_O_5_-induced bronchitis.

While much is known about signal transduction pathways that are activated by vanadium-induced oxidative stress, much less is know about genes that are regulated by these signaling pathways. In this study, we investigated V_2_O_5_-induced gene expression in cultured normal human lung fibroblasts using microarray analysis in order to gain a better understanding of the genes that mediate the pathogenesis of fibrosis.

## Methods

### Cell culture and materials

Normal adult human lung fibroblasts (ATCC 16 Lu) were purchased from American Type Culture Collection (Rockville, MD). Fibroblasts were seeded into 175 cm^2 ^plastic culture flasks and grown to confluence in 10% fetal bovine serum (FBS)/Dulbecco's modified Eagle's medium (DMEM), then trypsin-liberated, and seeded into 150 mm dishes. Confluent monolayers were rendered quiescent for 24 hrs in serum-free defined medium (SFDM) that consisted of Ham's F-12 medium with 0.25% BSA with an insulin/transferrin/selenium supplement. Cells were treated with 10 μg/cm^2 ^vanadium pentoxide, V_2_O_5 _(Aldrich Chemical, Milwaukee, WI) or SFDM and RNA was harvested from the fibroblast cultures at 1, 4, 8, 12 and 24 hrs post-treatment. We previously reported that this dose of V_2_O_5 _causes minimal cytotoxicity (<10% by lactate dehydrogenase assay) and yet induces H_2_O_2 _production, activates intracellular signaling pathways (e.g., MAP kinases), and upregulates growth factor production by human lung fibroblasts [11]. RNA from an SFDM control was harvested at each of these time points to normalize the V_2_O_5 _treatment at the same corresponding time point. Three replicate arrays were analyzed for SFDM and V_2_O_5 _treatment groups at each of the five time points tested.

### Microarray hybridizations and data analysis

Human lung fibroblast RNA was isolated using RNeasy columns (Qiagen, Valencia, CA). RNA quality was verified by spectrophotometry and gel electrophoresis using the Agilent 2100 Bioanalyzer (Agilent Technologies, Palo Alto, CA). Probe preparation and hybridization to the microarray was performed in the CIIT Gene Expression Core Facility using standard Affymetrix procedures. Double-stranded cDNA was synthesized from RNA using an oligo-dT24-T7. Biotinylated cRNA was synthesized from an aliquot of the cDNA template using the T7 RNA Transcript Labeling Kit (ENZO Diagnostics, Farmingdale NY). The labeled cRNA was then fragmented, hybridized to Affymetrix Human Genome U133A 2.0 arrays (Affymetrix, Santa Clara, CA), and stained using phycoerythrein-conjugated streptavidin (Molecular Probes, Eugene, OR). Gene expression results have been deposited in the National Center for Biotechnology Information (NCBI) Expression Omnibus database  [16](Accession Number GSE5339).

### Statistical analysis and data processing

The microarray data were preprocessed using RMA with a log base 2 (log_2_) transformation [17]. Statistical analysis of the data was performed in R using the affyImGUI package [18,19]. To identify genes with significant changes in expression following V_2_O_5 _exposure, all treatment groups were analyzed using a linear model with contrasts between untreated fibroblasts and V_2_O_5_-exposed fibroblasts at each time point. Genes from all of the five gene lists were combined for the final analysis. Probability values were adjusted for multiple comparisons using a false discovery rate of 5% (FDR = 0.05) [20]. Genes identified as statistically significant were subject to an additional filter by selecting only those genes that exhibited a ≥ 2-fold change from the untreated fibroblasts. Analysis of gene ontology (GO) categories was performed using NIH DAVID [21]. Statistical significance of the GO results was assessed using a hypergeometric test [21]. GO category hierarchy was obtained using AmiGO [22] and used to discard general categories from the DAVID analysis within the first three levels. Data for genes changed more than 2-fold were clustered using Cluster 3.0 [22] and visualized using the Mapletree Software program [24].

### Real Time quantitative RT-PCR

Total RNA from human lung fibroblasts was isolated using the Qiagen RNeasy Miniprep kit (Valencia, CA). One or two micrograms of total RNA was reverse transcribed at 48°C for 30 minutes using Multiscribe Reverse Transcriptase (Applied Biosystems, Foster City, CA) in 1 × RT buffer, 5.5 mM MgCl_2_, 0.5 μM of each dNTP, 2.5 μM of random hexamers, and 0.4 U/μL RNAse inhibitor in a volume of 100 μl. One hundred nanograms of the RT product was amplified using Taqman Gene Expression Assays on the Applied Biosystems 7700 Prism^® ^Sequence Detection System (Applied Biosytems, Foster City, CA). The PCR conditions and data analysis were performed according to the manufacturer's protocol described in User bulletin no.2, Applied Biosystems Prism 7700 Sequence Detection System. All samples were run in triplicate. Gene expression was measured by the quantitation of cDNA converted from mRNA corresponding to *VEGF*, *CTGF*, *HBEGF*, *IL8*, *CXCL9*, *CXCL10*, *PIPOX*, *OXR1*, *SOD2*, *STAT1*, *GAS1*, and *EGR1 *relative to the untreated control groups and normalized to 18S. 18S expression was not significantly changed in the microarrray experiment and therefore served as an appropriate housekeeping gene. Relative quantitation values (2−ΔΔCT
 MathType@MTEF@5@5@+=feaafiart1ev1aaatCvAUfKttLearuWrP9MDH5MBPbIqV92AaeXatLxBI9gBaebbnrfifHhDYfgasaacH8akY=wiFfYdH8Gipec8Eeeu0xXdbba9frFj0=OqFfea0dXdd9vqai=hGuQ8kuc9pgc9s8qqaq=dirpe0xb9q8qiLsFr0=vr0=vr0dc8meaabaqaciaacaGaaeqabaqabeGadaaakeaacqaIYaGmdaahaaWcbeqaaiabgkHiTiabfs5aejabfs5aejabboeadnaaBaaameaacqqGubavaeqaaaaaaaa@33ED@) were expressed as fold-change.

## Results

**Figure 1:**
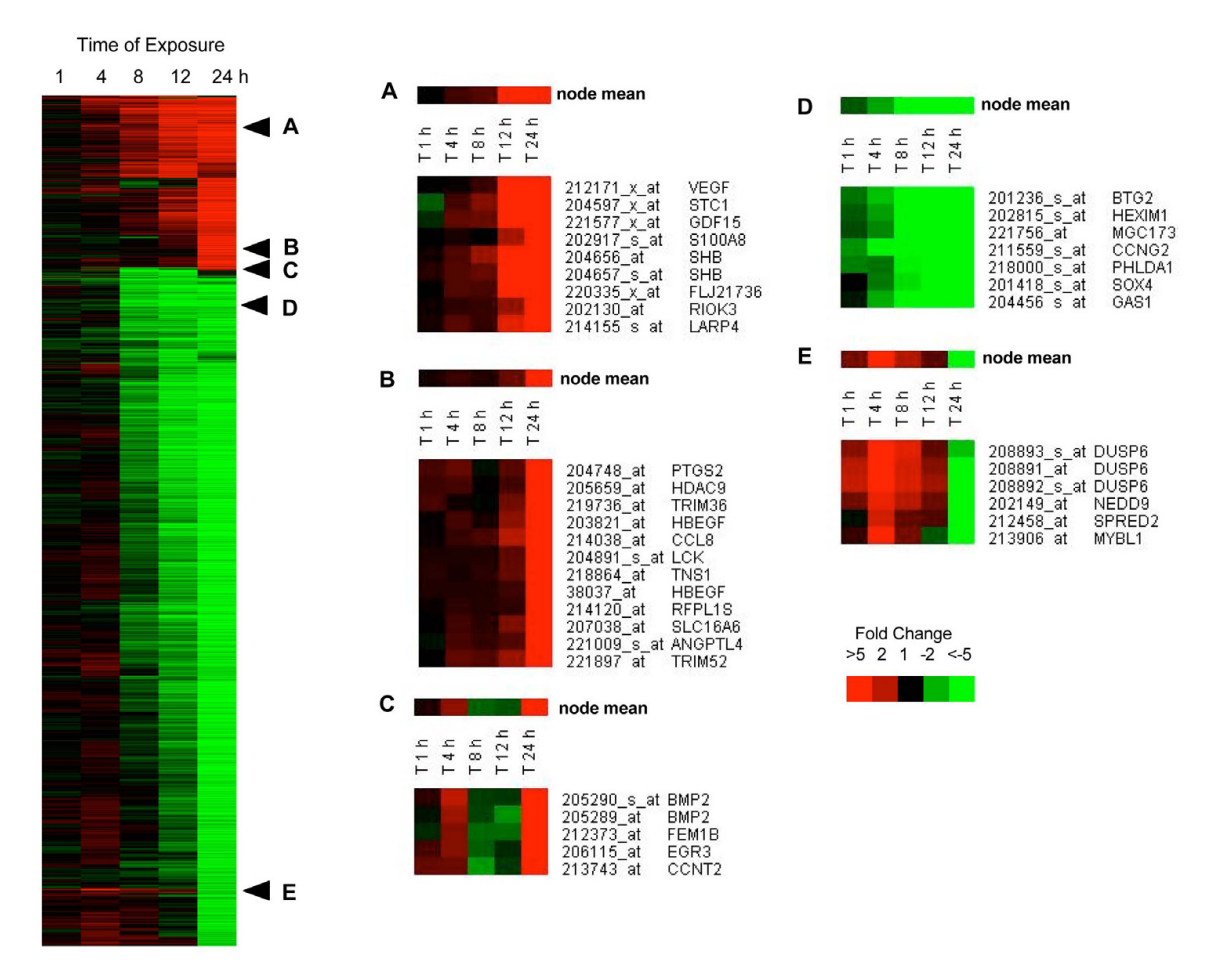
Heatmap showing hierarchical clustering of human lung fibroblast genes significantly induced (RED) or suppressed (GREEN) by V_2_O_5 _treatment. Gene expression in response to V_2_O_5 _was considered significant if p-value ≤ 0.05 and exhibited ≥ 2-fold change over untreated control. **Left panel**: All genes changed more than 2-fold. **Panels A and B**: Representative clusters of genes progressively induced. **Panel C**: Representative cluster of genes induced in a biphasic manner. **Panel D**: Representative cluster of suppressed genes. **Panel E**: Representative clusters of genes induced early then suppressed late.

**Figure 2:**
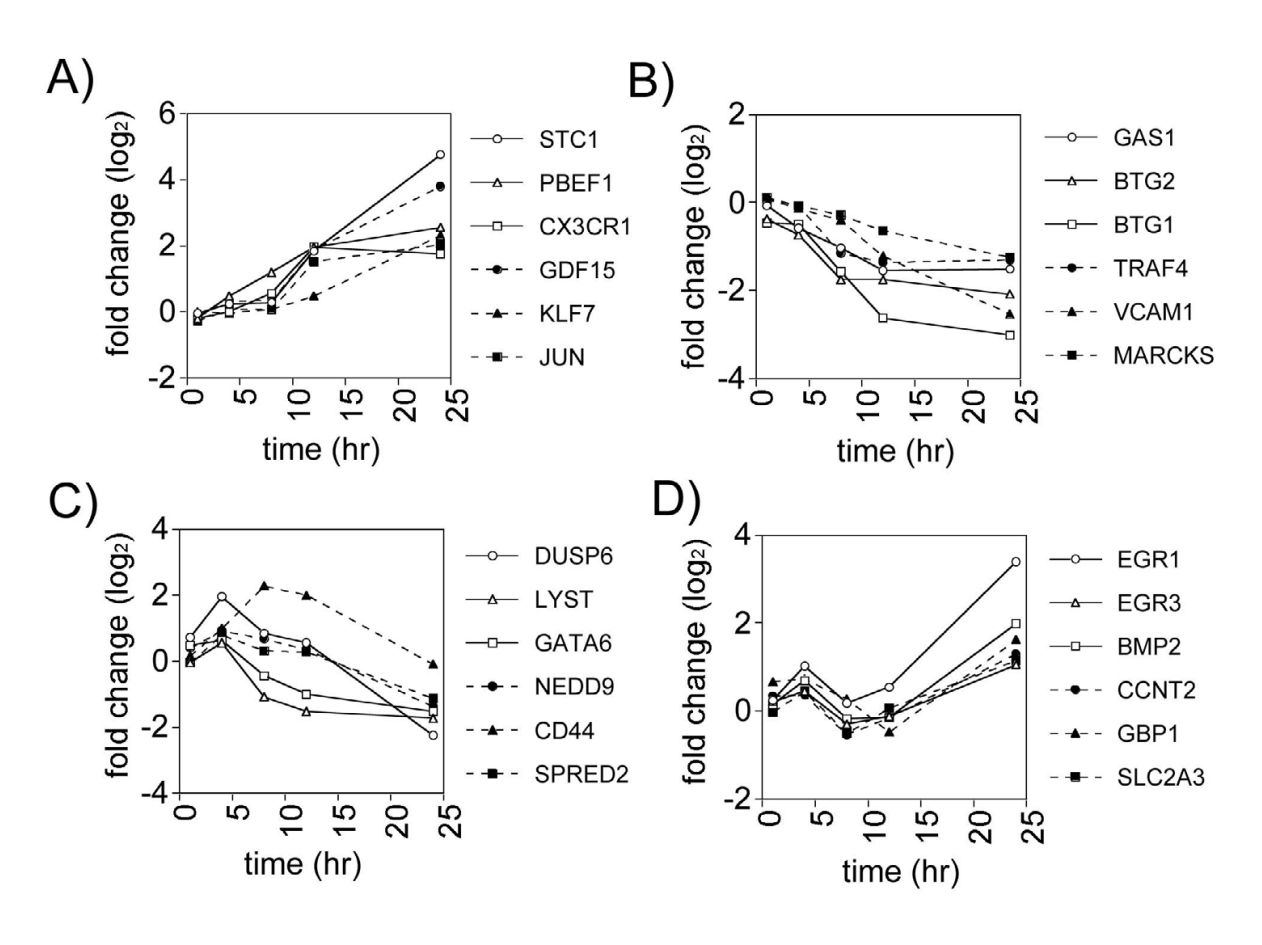
Gene expression profiles of selected genes of interest that fit one of four different temporal expression categories. Fold changes in gene expression over the time course of the experiment are shown on a log_2 _scale. **A) **Progressively induced genes, **B) **Progressively suppressed genes, **C) **Genes induced early and suppressed late, and **D) **Genes induced in a biphasic manner. The cellular localization and function of each of these genes are shown in Table 1.

Exposure of human lung fibroblasts to V_2_O_5 _resulted in significantly altered expression of over 1400 genes on the Affymetrix Human Genome U133A 2.0 Array. The majority of significantly changed genes were suppressed by V_2_O_5 _exposure over the 24 hr time course. Four major temporal patterns of gene expression were identified by hierarchical clustering analysis; progressively induced genes (Fig. 1A and 1B), genes that were induced in a biphasic manner (Fig. 1C), progressively suppressed genes (Fig. 1D) and early induced, late suppressed genes (Fig. 1E). Examples of genes from each of these temporal categories are shown in Fig. 2. The cellular localization and functions of selected genes from each of these categories is shown in Table 1.

**Table 1:** Temporal expression categories of selected genes significantly induced or suppressed by V_2_O_5 _exposure and their cellular localization and functions (See Fig. 2).

**Accession#**^a^	**Gene Symbol**	**Gene Name**	**Localization**	**Function**
**Progressively Induced Genes**				
Hs.25590	*STC1*	Stanniocalcin	Secreted	Cellular Metabolism
Hs.448611	*PBEF1*	Pre-B Cell Colony Enhancing Factor 1	Secreted	Inflammation
Hs.78913	*CX3CR1*	Chemokine (C-X3-C motif) Receptor 1	Membrane	Inflammation
Hs.515258	*GDF15*	Growth and Differentiation Factor-15	Secreted	Growth Inhibition
Hs.471221	*KLF7*	Kruppel-like factor 7	Nuclear	Transcriptional Regulation
Hs.525704	*JUN*	V-jun sarcoma virus 17 oncogene	Nuclear	Transcriptional Regulation
				
**Progressively Suppressed Gene**				
Hs.65029	*GAS1*	Growth Arrest Specific Gene 1	Nuclear	Growth Arrest and Apoptosis
Hs.519162	*BTG2*	B-Cell Translocation Gene 2	Nuclear	Growth Arrest
Hs.255935	*BTG1*	B-Cell Translocation Gene 1	Nuclear	Growth Arrest
Hs.8375	*TRAF4*	TNF Receptor-Associated Factor	Membrane	Inflammation/Immunity
Hs. 109225	*VCAM1*	Vascular Cell Adhesion Molecule 1	Membrane	Cell Adhesion
Hs.519909	*MARCKS*	Myristolated Alanine-rich C Kinase Substrate	Cytoplasmic	Cell Signaling
				
**Early Induced I/Late Suppressed Genes**				
Hs.298654	*DUSP6*	MAP kinase phosphatase 3	Cytoplasmic	Cell Signaling
Hs.532411	*LYST*	Lysosomal Trafficking Regulator Gene	Cytoplasmic	Cell Signaling
Hs.514746	*GATA6*	GATA6 Transcription Factor	Nuclear	Transcriptional Regulation
Hs.37982	*NEDD9*	Neural expressed Develop. down-regulated 9	Membrane	Cell Adhesion
Hs.502328	*CD44*	CD44 molecule (Indian blood group)	Membrane	Cell Signaling
Hs.59332	*SPRED2*	Sprouty-Related EVH Domain-2	Cytoplasmic	Cell Signaling
				
**Biphasic Induced Genes**				
Hs.326035	*EGR1*	Early Growth Response-1 Gene	Cytoplasmic/Nuclear	Transcriptional Regulation
Hs.534313	*EGR3*	Early Growth Response-3 Gene	Cytoplasmic/Nuclear	Transcriptional Regulation
Hs.73853	*BMP2*	Bone Morphogenic Protein-1	Secreted	Cell Differentiation
Hs.591241	*CCNT2*	Cyclin T2	Nuclear	Cell Cycle Regulation
Hs.62661	*GBP1*	guanylate-binding protein 1, IFN-inducible	Cytoplasmic	Antiviral Activitiy
Hs.419240	*SLC2A3*	Solute Carrier Family 2 (GLUT3)	Membrane	Metabolism

^a^Gene annotat ions are from NCBI .

An analysis of the biological processes (gene ontology categories) affected by V_2_O_5 _exposure in human lung fibroblasts was performed using the NIH DAVID program [21]. This analysis revealed that certain GO categories were unique to V_2_O_5_-induced genes, including *chemotaxis*, *inflammatory response*, *immune response*, and *cell-cell signaling *(Table 2). GO categories that were unique to suppressed genes included *ubiquitin cycle*, *cell cycle*, *DNA repair*, *nuclear transport*, and *programmed cell death*. A few categories such as *RNA processing *were common to induced and suppressed genes.

**Table 2:** Functional analysis of genes induced or suppressed by V_2_O_5 _in human lung fibroblasts.^a^

**GO ID**^b^	**GO Category**	**Genes**	**%**^c^	**P value**
**Induced Genes**				
0009605	response to external stimulus	32	8.47	1.43E-05
0006935	chemotaxis	13	3.44	6.96E-05
0009611	response to wounding	25	6.61	1.81E-04
0042221	response to chemical stimulus	23	6.08	2.51 E-04
0006950	response to stress	44	11.64	0.003553
0006928	cell motility	15	3.97	0.005005
0006396	RNA processing	19	5.03	0.005027
0008380	RNA splicing	11	2.91	0.007903
0006954	inflammatory response	13	3.44	0.011869
0008284	positive regulation of cell proliferation	10	2.65	0.013783
0006955	immune response	33	8.73	0.018616
0007267	cell-cell signaling	23	6.08	0.042107
				
**Suppressed Genes**				
0045449	regulation of transcription	298	19.34	3.61 E-25
0006512	ubiquitin cycle	81	5.26	1.16E-10
0006391	RNA processing	72	4.67	6.25E-10
0007049	cell cycle	113	7.33	4.42E-08
0006974	response to DNA damage stimulus	52	3.37	1.13E-07
0006295	DNA metabolism	94	6.10	2.23E-06
0006281	DNA repair	43	2.79	1.23E-05
0008380	RNA splicing	33	2.14	2.56E-05
0007243	protein kinase cascade	50	3.24	3.39E-05
0051301	cell division	31	2.01	2.71 E-04
0051169	nuclear transport	23	1.49	6.27E-04
0016310	phosphorylation	88	5.71	8.76E-04
0019538	protein metabolism	311	20.18	0.001149
0030518	steroid hormone receptor signaling pathway	13	0.84	0.001328
0050658	RNA transport	12	0.78	0.002917
0012501	programmed cell death	76	4.93	0.003907
0001558	regulation of cell growth	22	1.43	0.004779
0016568	chromatin modification	22	1.43	0.005351
0007259	JAK-STAT cascade	9	0.58	0.008321
0007050	cell cycle arrest	14	0.91	0.013090
0016055	Wnt receptor signaling pathway	18	1.17	0.020398
0015031	protein transport	65	4.22	0.034144
0008286	insulin receptor signaling pathway	6	0.39	0.039295
0007249	l-kappaB kinase/NF-kappaB cascade	18	1.17	0.042224

^a ^GO analysis performed using NIH DAVID .

^b ^Gene ontology ID numbers obtained from AmiGO .

^c^% of total induced or suppressed genes.

**Figure 3:**
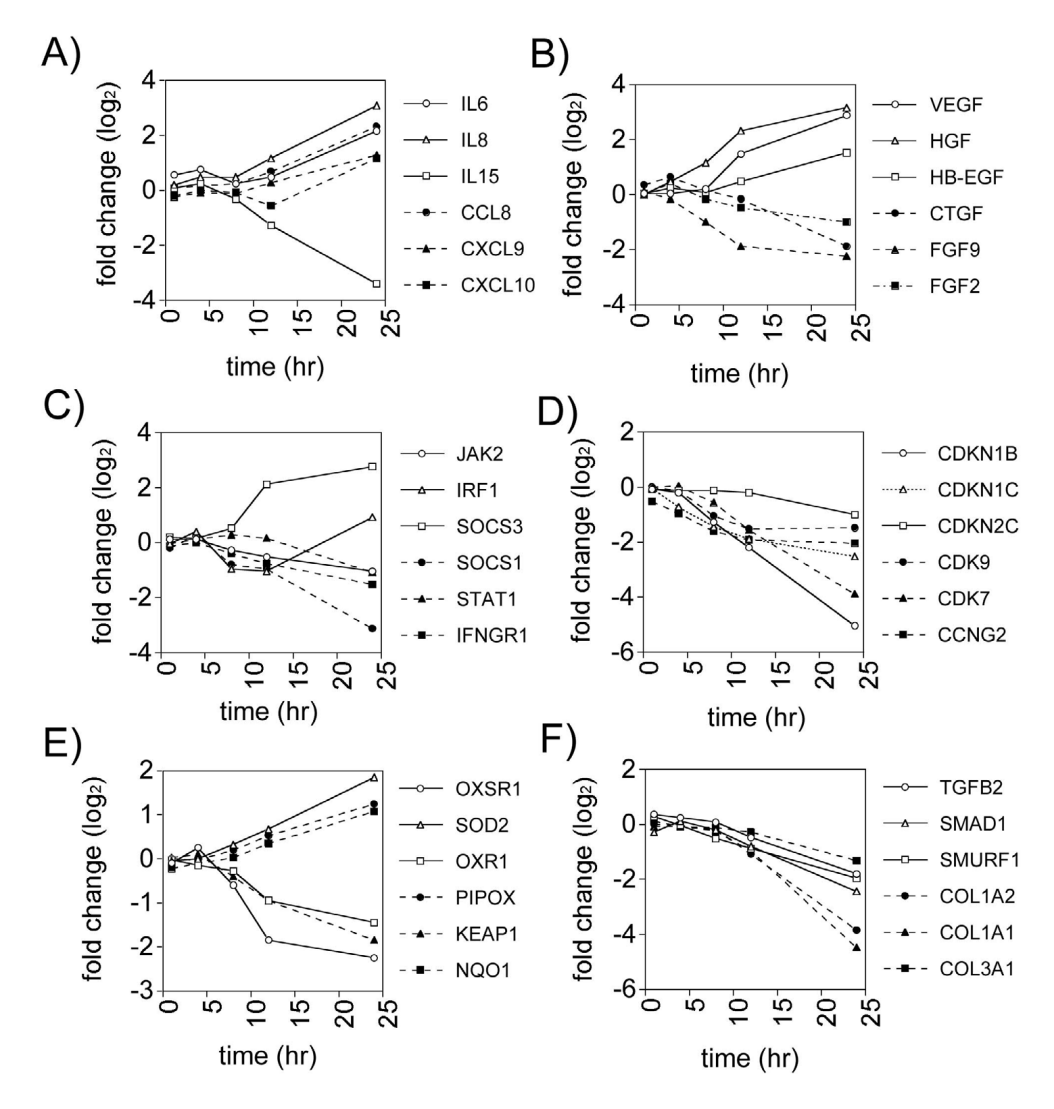
Gene expression profiles of selected genes for six functional categories. Fold changes in gene expression over the time course of the experiment are shown on a log_2 _scale. **A) **Cytokines and Chemokines, **B) **Growth Factors, **C) **STAT Signaling, **D) **Cell Cycle Regulation, **E) **Oxidative Stress, and **F) **TGF-β Signaling. The cellular localization and function of each of these genes are shown in Table 3.

While analysis of GO biological processes was useful in assessing the overall numbers of significantly changed genes in various functional categories, we selectively grouped genes that have been shown to play important roles in various aspects of tissue injury, repair, and remodeling. These categories included A) *cytokines and chemokines*, B) *growth factors*, C) *STAT signaling*, D) *cell cycle regulation*, E) *oxidative stress*, and F) *TGF-β signaling *(Fig. 3). The functions and cellular localization of representative genes from each of these categories is shown in Table 3. A number of cytokines and chemokines were induced over the time course, including *IL8*, *IL-6*, *CCL8*, *CXCL9*, and *CXCL10*, while *IL15 *was suppressed in a time-dependent manner (Fig. 3A). *VEGF*, *HGF*, and *HBEGF *were progressively induced, while *FGF2 *and *FGF9 *were suppressed (Fig. 3B). *CTGF *was induced early (4 hrs) and suppressed late. Members of the STAT signaling pathway were differentially regulated (Fig. 3C). IRF-1 was induced in a biphasic manner. *SOCS3 *was progressively induced over the time course, while *SOCS1 *and *IFNGR *were progressively suppressed. Genes encoding cell cycle regulation were mainly suppressed, including *CDKN1B *and *CDKN1C*, which function to inhibit cell cycle progression (Fig. 3D). Oxidative stress genes were differentially regulated. In particular, *SOD2 *and *PIPOX*, which function in peroxide generation, were progressively induced (Fig. 3E). *OXR1 *and *OXSR1*, which are protective against oxidative stress, were suppressed. Genes involved in TGF-β signaling and collagen deposition were suppressed, including *TGFB2*, *SMAD1*, *SMURF1*, *COL1A1*, *COL1A2*, and *COL3A1 *(Fig. 3F).

**Table 3:** Cellular localization and functions of genes regulated by V_2_O_5 _grouped by functional categories (See Fig. 3).

**Accession#**^a^	**Gene Symbol**	**Gene Name**	**Localization**	**Function**
**Cytokines and Chemokines**				
Hs.512234	*IL6*	lnterleukin-6 (interferon beta2)	Secreted	Inflammation
Hs.624	*IL8*	lnterleukin-8	Secreted	Neutrophil Chemotaxis
Hs. 168132	*IL15*	lnterleukin-15	Secreted	T Lymphocyte Proliferation
Hs.271387	*CCL8*	CC Chemokine Ligand 8	Secreted	Neutrophil Chemotaxis
Hs.77367	*CXCL9*	Chemokine (C-X-C motif) Ligand 9 (Mig)	Secreted	Inflammation
Hs.632586	*CXCL10*	Chemokine (C-X-C motif) Ligand 10 (IP-10)	Secreted	Inflammation
				
**Growth Factors**				
Hs.73793	*VEGF*	Vascular Endothelial Cell Growth Factor	Secreted	Endothelial Cell Growth
Hs.396530	*HGF*	Hepatocyte Growth Factor	Secreted	Epithelial Cell Growth
Hs.799	*HBEGF*	Heparin-Binding EGF-like Growth Factor	Membrane/Secreted	Fibroblast Growth
Hs.591346	*CTGF*	Connective Tissue Growth Factor	Secreted	Collagen Synthesis
Hs.111	*FGF9*	Fibroblast Growth Factor-9	Membrane/Secreted	Fibroblast Growth
Hs.284244	*FGF2*	Fibroblast Growth Factor-2	Membrane/Secreted	Fibroblast Growth
				
**STAT Signaling**				
Hs.591081	*JAK2*	Janus Activated Kinase-2	Membrane	STAT Phosphorylation
Hs.436061	*IRF1*	Interferon-Regulatory Factor-1	Cytoplasmic/Nuclear	Transcriptional Regulation
Hs.527973	*SOCS3*	Suppressor of Cytokine Signaling-3	Cytoplasmic	Cell Signaling
Hs.50640	*SOCS1*	Suppressor of Cytokine Signaling-1	Cytoplasmic	Cell Signaling
Hs.470943	*STAT1*	Signal Transducer Activator of Transcription	Cytoplasmic	Growth Arrest and Apoptosis
Hs.520414	*IFNGR1*	Interferon Gamma Receptor- 1	Membrane	Cell Signaling
				
**Cell Cycle Regulation**				
Hs.238990	*CDKN1B*	Cyclin-Dependent Kinase lnhbitior-1B (Kip1)	Nuclear	Cell Cycle Arrest
Hs. 106070	*CDKN1C*	Cyclin-Dependent Kinase lnhibitor-1C (Kip2)	Nuclear	Cell Cycle Arrest
Hs.525324	*CDKN2C*	Cyclin-Dependent Kinase lnhibitor-2C	Nuclear	Cell Cycle Arrest
Hs.557646	*CDK9*	Cyclin-Dependent Kinase-9	Nuclear	Transcriptional Regulation
Hs. 184298	*CDK7*	Cyclin-Dependent Kinase-7	Nuclear	Transcriptional Regulation
Hs. 13291	*CCNG2*	Cyclin G2	Nuclear	Cell Cycle Arrest
				
**Oxidative Stress**				
Hs.475970	*OXSR1*	Oxidative Stress Response 1	Cytoplasmic	Intracellular Kinase
Hs.487046	*SOD2*	Superoxide Dismutase 2 (SOD2)	Cytoplasmic	Peroxide Generation
Hs. 148778	*OXR1*	Oxidative Resistance 1	Cytoplasmic	Anti-Oxidant
Hs.462585	*PIPOX*	Pipecolic Acid Oxidase	Cytoplasmic	Peroxide Generation
Hs.465870	*KEAP1*	Kelch-like ECH-associated protein 1	Cytoplasmic	Redox Homeostasis
Hs.406515	*NQO1*	NAD(P)H:quinone oxidoreductase 1	Cytoplasmic	Redox Homeostasis
				
**TGF-beta Signaling and Collagen**				
Hs. 133379	*TGFB2*	Transforming Growth Factor beta-2	Secreted	Matrix Synthesis, Immunity
Hs.519005	*SMAD1*	mothers against DPP homolog 1	Cytoplasmic	Cell Signaling
Hs. 189329	*SMURF1*	Smad Ubiquitin Regulatory Factor-1	Cytoplasmic	Cell Signaling
Hs.489142	*COL1A2*	Collagen 1A2	Secreted	Structural Protein
Hs. 172928	*COL1A1*	Collagen 1A1	Secreted	Structural Protein
Hs.443625	*COL3A1*	Collagen 3A1	Secreted	Structural Protein

^a^Gene annotations are from NCBI .

**Figure 4:**
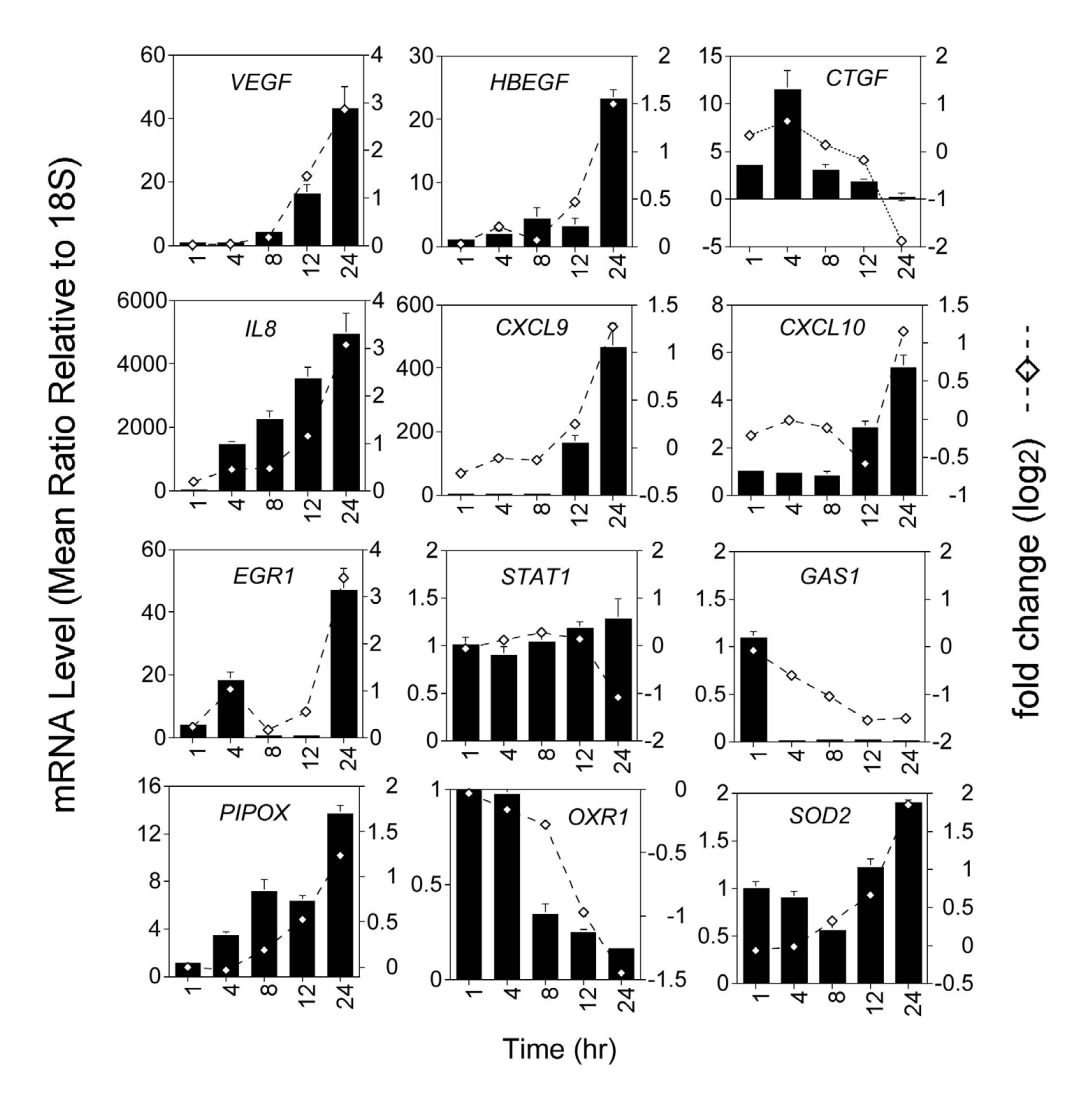
Validation of selected genes by Taqman quantitative RT-PCR. RNA was isolated from human lung fibroblasts treated with 10 μg/cm^2 ^V_2_O_5 _at the indicated time points and RT-PCR performed as described in Methods. Three genes from four categories were validated; growth factors (top row: *VEGF*, *HBEGF*, *CTGF*), chemokines (second row: *IL8*, *CXCL9*, *CXCL10*), transcription factors (third row: *Egr1*, *STAT1*, *GAS1*), and oxidative stress genes (bottom row: *PIPOX*, *OXR1*, *SOD2*). The data for each gene was normalized against 18S housekeeping gene and expressed as the mean ratio. Data are representative of at least two replicate experiments and expressed as the mean ± sem of triplicate dishes of cells. The temporal pattern of each V_2_O_5_-altered gene validated by RT-PCR is compared with the result obtained from the microarray experiment (open diamonds).

Taqman quantitative real time RT-PCR was used to validate a dozen selected genes that were induced or suppressed by V_2_O_5 _exposure. We chose to validate 3 genes from each of the following categories (*growth factors, chemokines, transcription factors, oxidative stress*) that appear to have important roles in inflammation, repair, or fibrosis. The results obtained with Taqman quantitative RT-PCR closely mirrored the patterns of temporal induction or suppression observed in the microarray experiment (Fig. 4).

## Discussion

**Figure 5:**
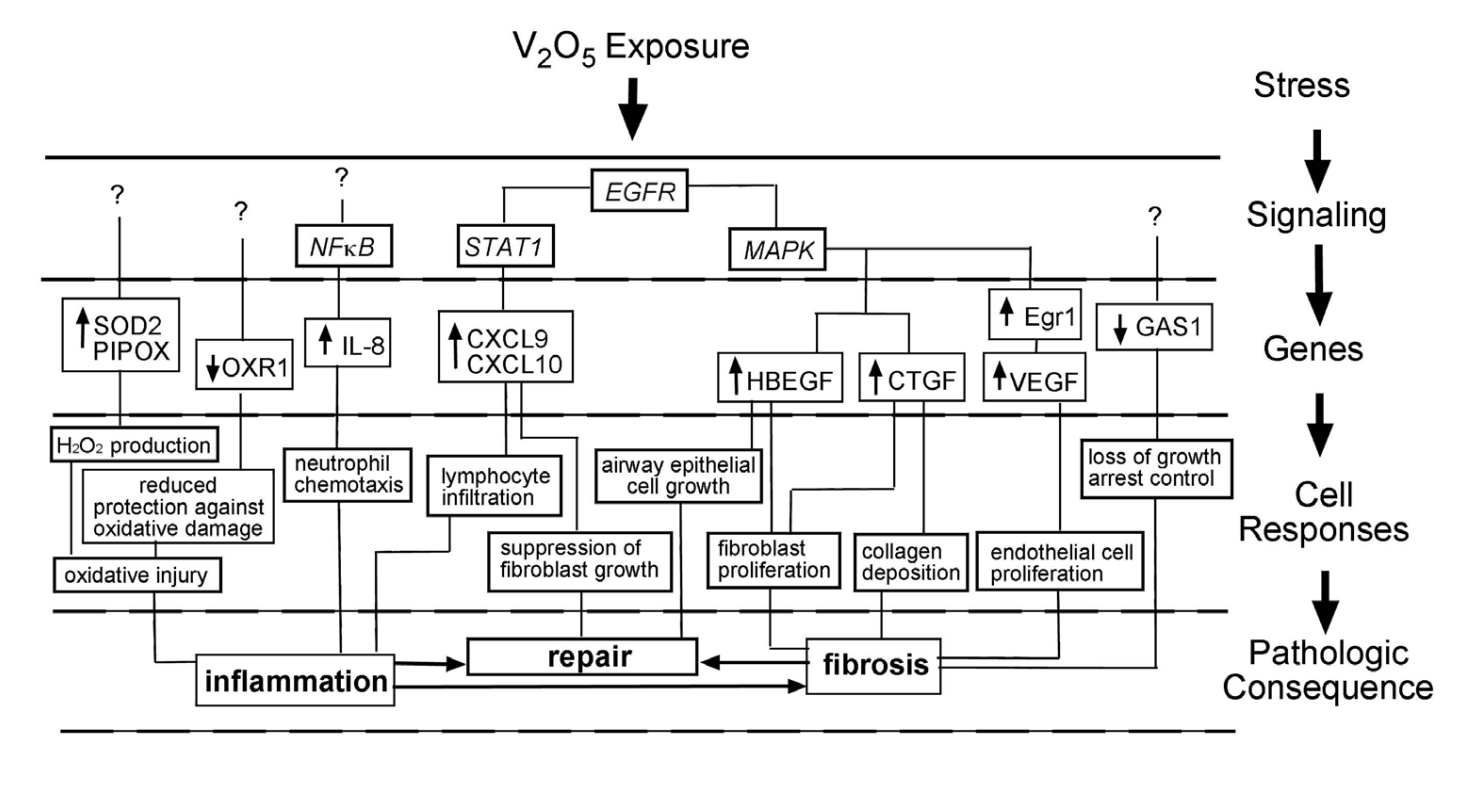
Illustration showing postulated roles of selected V_2_O_5_-induced or -suppressed genes in the context of upstream cell signaling events and downstream cell responses and pathologic consequences. All genes shown were validated by quantitative RT-PCR (see Fig. 4).

Occupational exposure to vanadium oxides has been associated with an increased incidence of obstructive airway disease and a reduction in lung function [1]. In the present study, we investigated the temporal expression of genes in normal human lung fibroblasts exposed V_2_O_5_. We previously reported that 10 μg/cm^2 ^V_2_O_5_, the same dose used in our microarray experiment, causes minimal cytotoxicity (<10%) to fibroblasts or epithelial cells over a 24 hr time period [10,11]. This concentration of V_2_O_5 _also causes several well-defined phenotypic changes in lung fibroblasts including a marked increase in H_2_O_2 _by fibroblasts [11], phosphorylation of the signal transducer and activator of transcription (STAT-1) [8], and increased expression of heparin-binding EGF-like growth factor, HBEGF [11]. Our current study identified genes regulated by V_2_O_5 _that could play potentially important roles in oxidative stress, inflammation, growth, and apoptosis during V_2_O_5_-induced lung injury, remodeling and repair. Moreover, our investigation suggests that fibroblasts play an important role in orchestrating the responses of other pulmonary cell types, including neutrophils, airway epithelial cells, lymphocytes, and endothelial cells. The postulated roles of selected genes that were validated by RT-PCR in mediating V_2_O_5_-induced inflammation, repair, and fibrosis are illustrated in Fig. 5.

A variety of genes encoding cytokines and chemokines were induced or suppressed by V_2_O_5_. For example, V_2_O_5 _induced *IL8 *and *IL6*, which play important roles in acute inflammation. We validated the strong induction of *IL8 *mRNA by RT-PCR. Vanadium rich oil fly ash has been reported to increase *IL8 *and *IL6 *mRNA and protein expression in normal human airway epithelial cells [25,26]. Moreover, workers exposed to vanadium-rich fuel oil ash have increased IL8 protein in nasal fluid [27]. Chemokines induced by V_2_O_5 _could play important roles in the immune response. Notably, V_2_O_5 _induced *CXCL9 *(*Mig*) and *CXCL10 *(inducible protein-10), both of which were validated by RT-PCR. *CXCL9 *and *CXCL10 *are STAT1-dependent chemokines that function in the recruitment of lymphocytes [28]. We previously showed that V_2_O_5 _activates STAT1 in lung fibroblasts [8] and mice deficient in STAT1 are susceptible to pulmonary fibrosis [29]. Moreover, we have observed intratracheal V_2_O_5 _exposure in rats causes lymphocytic accumulation surrounding airways and small blood vessels, as well causing proliferation of lymphocytes within the bronchus-associated lymphatic tissue adjacent to large airways [30]. It is possible that STAT1-dependent induction of *CXCL9 *and *CXCL10 *could be a mechanism for lymphocyte accumulation around airways and blood vessels following lung injury by V_2_O_5_.

Polypeptide growth factors have a variety of functions in airway remodeling that occurs after metal-induced lung injury. Our genomic analysis identified several growth factors that were validated by RT-PCR. Each of these genes had a different temporal pattern of expression. First, vascular endothelial cell growth factor (*VEGF*) was progressively induced after V_2_O_5 _treatment. Li and coworkers showed that vanadium induces the expression of *VEGF *in a mouse epithelial cell line through the activation of ERK [31]. *VEGF *promotes angiogenesis by stimulating the proliferation of vascular endothelial cells and fibroblasts [32]. Our data suggest that fibroblasts could function to promote the formation new blood vessels in V_2_O_5_-induced airway fibrotic lesions by signaling endothelial cells via VEGF protein or it is possible that secreted VEGF could stimulate fibroblast replication. Second, *HBEGF *gene expression was increased in a biphasic manner. *HBEGF *functions both in fibroblast mitogenesis and in epithelial repair [10,11]. Third, connective tissue growth factor (*CTGF*) was increased transiently in human lung fibroblasts and then suppressed. We have also reported that V_2_O_5 _increases *CTGF *mRNA in the lungs of rats exposed by intratracheal instillation [30]. The temporal differences in the expression of *VEGF*, *HBEGF*, and *CTGF *after V_2_O_5 _treatment remain unclear. We have reported that the early induction of *HBEGF *is due to peroxide dependent activation of MAP kinases [11]. We have also observed that V_2_O_5_-induced *CTGF *expression requires MAP kinases (Ingram and Bonner, unpublished observation). The late induction of *HBEGF *and *VEGF *could be due to the delayed induction of a transcriptional regulator gene that is increased in response to V_2_O_5_-induced oxidative stress. One such transcriptional regulator that serves as a master switch for growth factor induction is the early growth response (*EGR1*) gene. *EGR1 *was significantly induced at 4 and 24 hr following V_2_O_5 _treatment in both microarray and RTPCR experiments. *EGR1 *is induced by a variety of factors including cellular stress and functions as a transcriptional regulator to increase the expression of growth factor genes such as *VEGF *[33].

Other growth response genes, including the growth arrest specific (*GAS1*) gene and Bcell translocation genes (*BTG1 *and *BTG2*), were progressively suppressed in a time dependent manner after V_2_O_5 _exposure. *BTG1*, *BTG2*, and *GAS1 *are all anti-mitogenic factors that mediate growth arrest of fibroblasts [34-36]. Cyclin-dependent kinase inhibitors, *CDKN1B *p27(Kip1) and *CDKN1C *p57(Kip2), were also progressively suppressed. These two kinase inhibitors mediate growth arrest and serve as tumor suppressors [37,38]. Overall, our data suggests that V_2_O_5 _stimulates the growth and survival of fibroblasts by suppressing genes encoding anti-mitogenic factors (*GAS1*, *BTG2*, *CDKN1B*, and *CDKN1C*). In particular, our RT-PCR results validated *GAS1 *suppression in V_2_O_5_-exposed fibroblasts. While the increased expression of growth factors (i.e., *VEGF*, *HBEGF*, *CTGF*) by fibroblasts exposed to V_2_O_5 _is likely important in promoting fibroblast growth and survival, the reduced expression of *GAS1 *by V_2_O_5 _could be equally important in promoting fibroblast replication and survival. Moreover, V_2_O_5 _progressively suppressed *GAS1 *over the entire time course of the experiment, indicating sustained loss of growth arrest control when growth factors such as *VEGF*, *HBEGF*, and *CTGF *were maximally induced.

We found that V_2_O_5 _induced or suppressed a number of genes that are involved in oxidative stress. Vanadium compounds have been reported to activate several transcription factors and induce the release of inflammatory mediators through the generation of H_2_O_2 _[13,14,8]. Also, we previously reported that human lung fibroblasts exposed to V_2_O_5 _release micromolar amounts of H_2_O_2 _*in vitro *12 to 18 hrs after V_2_O_5 _exposure [11]. Two genes encoding peroxide-generating enzymes, *SOD2 *and *PIPOX*, were validated by RT-PCR. *SOD2 *was progressively increased over the 24 hr time course of V_2_O_5 _exposure. *SOD2 *serves as a major protective anti-oxidant defense enzyme that converts superoxide anion to H_2_O_2 _[39]. V_2_O_5 _undergoes redox chemistry to generate superoxide anion, so it is possible that *SOD2 *plays a role in reducing V_2_O_5_-induced lung injury. L-pipecolate oxidase (*PIPOX*), a peroxisomal oxidase, was also progressively induced by V_2_O_5_. *PIPOX *utilizes molecular oxygen as a substrate with H_2_O_2 _as a product [40]. While V_2_O_5 _induces genes that generate peroxide (*SOD2*, *PIPOX*), we also validated suppression of the oxidative resistance gene (*OXR1*). Volkert and colleagues discovered the human *OXR1 *gene using a functional genomics approach in a search for genes that function in protection against oxidative damage [41]. While *OXR1 *is protective against oxidative stress, the precise function of this gene is not well understood. Because *OXR1 *is protective against oxidative injury, suppression of this gene could contribute to V_2_O_5_-induced oxidative stress. Also, the temporal suppression of *OXR1 *occurs as *PIPOX *(a pro-oxidative stress gene) is temporally induced.

V_2_O_5 _causes airway fibrosis in rats *in vivo*, and it is well known that increased collagen production defines the fibrotic lesion [4]. TGF-β is an essential mediator of collagen production by fibroblasts. Our results showed that *TGFB2*, along with its associated signaling intermediates *SMAD1 *and *SMURF1*, were all progressively suppressed by V_2_O_5_. Moreover, several major collagen genes (*COL1A2*, *COL1A1*, *COL3A1*) were suppressed as well. These data indicate that V_2_O_5 _does not directly stimulate fibroblasts to deposit collagen. Instead, it is likely that TGF-β or other factors signals produced by neighboring pulmonary cell types to increase collagen production. TGF-β mRNA is increased in the lungs of rats treated with V_2_O_5_. Therefore, during V_2_O_5_-induced fibrogenesis fibroblasts do not appear to be effectors of their own collagen deposition, but likely require other cell types (e.g., macrophages) as a source of TGF-β.

While we used lung fibroblasts in our study, it is highly relevant to consider the effect of V_2_O_5 _exposure on gene expression by other lung cell types, including epithelial cells. Li and colleagues used microarray analysis to investigate gene expression changes in human bronchial epithelial cells exposed to vanadium or zinc and identifed a small set of genes that could be used as biomarkers for discriminating vanadium from zinc [42]. They also reported that *IL8 *and *PTGS2 *(COX-2) were induced several-fold by vanadium but not by zinc. *IL8 *and *PTGS2 *were also strongly induced in human lung fibroblasts by vanadium in our study. In fact, we previously reported that COX-2 null mice are susceptible to V_2_O_5_-induced lung fibrosis, which emphasized an important protective role for the *PTGS2 *gene during fibrogenesis [43].

## Conclusion

A variety of genes were induced or suppressed in normal human lung fibroblasts by vanadium pentoxide (V_2_O_5_) that appear to have important functions in inflammation, fibrosis and repair. Our data suggest that both the induction of genes that mediate cell proliferation and chemotaxis (*VEGF*, *CTGF*, *HBEGF*), as well as suppression of genes involved in growth arrest and apoptosis (*GAS1*), is important to the lung fibrotic reaction to V_2_O_5_. The induction of interferon-inducible, STAT1-dependent chemokines (*CXCL9 and CXCL10*) could contribute to both suppression of fibroblast proliferation and lymphocyte accumulation. The strong induction of *IL8 *likely contributes to neutrophilic inflammation. An increase in peroxide-generating enzymes (*PIPOX*, *SOD2*) is consistent with H_2_O_2 _production by V_2_O_5_, while the reduced expression of protective oxidative response genes (e.g., *OXR1*) could further contribute to oxidative damage. Overall, our study reveals a wide variety of candidate genes that could mediate V_2_O_5_-induced airway remodeling after occupational and environmental exposures.

## Abbreviations

V_2_O_5_, vanadium pentoxide; STAT-1, signal transducer and activator of transcription; GAS1, growth arrest specific gene; VEGF, vascular endothelial cell growth factor; CTGF, connective tissue growth factor; CXCL10, Chemokine (C-X-C motif) ligand 10; HB-EGF, heparin-binding epidermal growth factor-like growth factor; PTGS-2, prostaglandin synthase 2; OXR1, oxidative resistance gene; SOD2, superoxide dismutase-2; PIPOX, L-pipecolate oxidase.

## Competing interests

The author(s) declare that they have no competing interests.

## Authors' contributions

JLI and JCB designed the experiments, performed the data analysis, and drafted the manuscript. JLI, AAM, EAT, JBM, and DGW performed cell culture, RNA isolation, and validated changes in selected genes by Taqman quantitative real-time RT-PCR. LJP performed with microarray hybridizations. RST performed statistical analysis on the microarray data. All authors read and approved the final manuscript.
